# Cardiovascular risk reduction with integrated care: results of 8 years follow up

**DOI:** 10.1186/s12875-023-02010-y

**Published:** 2023-03-08

**Authors:** Geert H. J. M. Smits, Sander van Doorn, Michiel L. Bots, Monika Hollander

**Affiliations:** 1 Julius Centre for Health Sciences and Primary Care, University Medical Centre Utrecht, Utrecht University, Utrecht, The Netherlands; 2 Primary Care Group PoZoB, Bolwerk 10-14, 5509 MH Veldhoven, The Netherlands

**Keywords:** Primary health care, Cardiovascular risk management, Integrated care, Practice nurse

## Abstract

**Background:**

Care groups organize integrated cardiovascular risk management programs in primary care for high risk patients. Results of long term cardiovascular risk management are scarce. The aim was to describe changes in low density lipoprotein cholesterol, systolic blood pressure and smoking between 2011 and 2018 in patients participating in an integrated program for cardiovascular risk management organized by a care group in the Netherlands.

**Aim:**

To explore whether long-term participation in an integrated cardiovascular risk management program could lead to the improvement of 3 important risk factors for cardiovascular disease.

**Methods:**

A protocol was developed for delegated practice nurse activities. A multidisciplinary data registry was used for uniform registration. The care group organized annual education for general practitioners and practice nurses on cardiovascular topics and regular meetings for practice nurses only to discuss complex patient cases and implementation issues. From 2015 onwards, the care group started with practice visitations to discuss performance and support practices with organizing integrated care.

**Results:**

In patients eligible for primary prevention as well as for secondary prevention similar trends were observed: lipid modifying and blood pressure lowering medication increased, mean low density lipoprotein cholesterol and mean systolic blood pressure decreased, patients on target for low density lipoprotein cholesterol and systolic blood pressure increased and the proportion of non-smokers with both low density lipoprotein cholesterol and systolic blood pressure on target increased. Improved registration between 2011 and 2013 was partly responsible for the sharp increase of patients on target for low density lipoprotein cholesterol and systolic blood pressure.

**Conclusion:**

In patients participating in an integrated cardiovascular risk management program, we saw annual improvements in 3 important cardiovascular risk factors between 2011 and 2018.

## Introduction

Cardiovascular disease (CVD) accounts for 45% of deaths and 64 million disability adjusted life years (DALY) in Europe [1], with reduced quality of life in patients after a myocardial infarction and stroke [2, 3]. The occurrence of CVD can be largely attributed to modifiable risk factors such as elevated blood pressure, unfavorable lipids, obesity, smoking, physical inactivity, unhealthy diet and alcohol intake of which it has been suggested that adjustment to normal levels could prevent 80–90% of all myocardial infarctions and ischemic strokes [4, 5]. Addressing these risk factors with a multifactorial and multidisciplinary approach is recommended by national and international guidelines [6, 7]. With a long tradition in programmatic prevention and a longstanding relationship with patients and their families, general practitioners (GPs) play a key-role in a multidisciplinary approach for the prevention of CVD. Both patients and GPs endorse the usefulness of prevention in primary care [8]. The National Guideline for cardiovascular risk management (CVRM) indicates how to identify patients with an increased risk of CVD and advised on cardiovascular risk reduction with lifestyle improvements and drug therapy [6]. Moreover, the guideline provides advice on task delegation, such as to the practice nurse (PN), a registered nurse with an additional 1 year training. Having their own consultation hours, PNs perform protocolized programmatic CVRM care, focusing on life style and treatment of blood pressure and cholesterol. Involvement of trained nurses has shown to be equivalent to physician care regarding health outcomes for patients, process of care, referrals to specialists and costs [9]. Additionally, compared with GPs, nurse-delivered CVRM in primary care led to better outcome measures on systolic blood pressure, total cholesterol and LDL-cholesterol after 1 year [10].

Surveys carried out in several European countries showed poor implementation of international CVRM guidelines in primary care and disappointing rates of cardiovascular risk factor control [11, 12]. A comprehensive approach is generally needed to overcome many barriers for following guidelines and treatment targets [13]. Successful introduction and implementation of a guideline depends highly on a rigorous preparation and evaluation [14]. Between 2005 and 2010 in the Netherlands, many GPs organized themselves in primary care groups who became responsible for the organization and provision of high-quality, guideline based CVRM care [15]. Care groups negotiate with regional health insurance organizations about structural renumeration for the organization of integrated CVRM care. Practices benefit from negotiated renumeration, care group support with implementation and regular education for GPs and PNs, implicating that all practices are affiliated to the care group. The care group receives a negotiated renumeration from health insurance companies for every participating patient in the care program. In 2020, 86 care groups offered CVRM care to 15,5 million registered patients in the Netherlands. In the Eindhoven region, south-east of the Netherlands, 3 care groups (Praktijk ondersteuning Zuid oost Brabant: PoZoB, Stichting Gezondheidscentra Eindhoven: SGE and De Ondernemende Huisarts: DOH) with around 300 affiliated GPs offer integrated CVRM care. PoZoB implemented integrated CVRM care in 137 practices (200 GPs) between April 1^st^ 2010 and January 1^st^ 2013. Since long term results of its effect on cardiovascular risk factors are scarce, we describe the changes in LDL-cholesterol, systolic blood pressure (SBP) and smoking status of patients enrolled in a CVRM care program between 2011 and 2018.

## Methods

### Design

The CVRM implementation program was designed as a dynamic observational cohort study, with a run in period (2010–2015) for optimizing data collection and protocol development, aiming at improvements in registration and outcomes identical for all participating practices. From 2015 to 2018 the care group focused on visitation of individual practices to discuss practice organization and outcomes of integrated care.

### Study population

The study was performed in the region Eindhoven, south-eastern part of the Netherlands, in 145 general practices affiliated to the primary care group PoZoB. The care group covers rural, suburban and urban practices similar to other parts of the Netherlands and therefore can be considered as representative. Between 2010 and 2013, 137 practices (406,119 registered patients) followed a stepwise implementation for integrated care and another 8 practices started implementation between 2013 and 2015. Eligibility for participation in integrated CVRM care was based on in- and exclusion criteria given in Table 1. Details of the stepwise implementation have been described elsewhere [16].

**Table 1:** Criteria for participation in the CVRM program ^a^

Inclusion criteria for patients eligible for primary prevention
● A 10 year cardiovascular mortality risk > 5%, based on the SCORE table from the 2006 CVRM guidelines of the Dutch Society of General Practice [6]
● Prescription of blood pressure lowering or lipid modifying drugs in men aged ≥ 55 years and women aged ≥ 60 years
● Systolic blood pressure > 180 mm Hg and/or total cholesterol > 8 mmol/l ever measured, independent of the 10 year mortality risk
● The patient is primarily treated in primary care and aged 18 years or above
Inclusion criteria for patients eligible for secondary prevention:
● Documented previous ischemic or atherosclerotic heart disease (myocardial infarction and angina pectoris), heart failure, atrial fibrillation, aneurysm of the abdominal aorta, peripheral arterial disease, transient ischemic attack, ischemic or hemorrhagic stroke, chronic kidney disease
● The patient is primarily treated in primary care and aged 18 years or above
Exclusion criteria for both groups were:
● Primarily treated for cardiovascular disease risk by a specialist in a hospital or at an outpatient clinic
● Diabetes mellitus (patients receive cardiovascular risk management in a diabetes care program)
● Patients younger than 18 years

^a^CVRM program: cardiovascular risk management program

### Interventions between 2010 and 2018

#### Registration

A multidisciplinary registry for integrated care (Care2U), set up from April 1^st^ 2010 onwards, collected data mainly from PNs, but other primary health care workers like dieticians and physiotherapists could add data as well. Data in Care2U automatically ended up in the GPs Electronic Health Record (EHR) and were visible for individual practices in real time. Laboratory test results ended up automatically in Care2U. Smoking status and all SBP measurements taken in one year were registered in Care2U, with the last measured SBP value visible in the data overview of every individual practice. Due to linking problems of Care2U with 8 different EHR systems, data from 2010 were incomplete but registration improved significantly in 2011 and 2012. In 2011, Care2U-data of 34,628 participating patients (mean age 67 years, 53% women, 29% with a previous CVD) was available, increasing to data of 48,397 patients (mean age 70 years, 51% women, 44% with a previous CVD) in 2018. Prevalence increased annually due to new affiliating practices after 2013, increased incidence of patients eligible for primary prevention and referrals from the specialist.

#### Integrated CVRM care

After assessment for eligibility patients started with life style improvements and drug therapy. If necessary referral to another health care professional was made, such as a dietician, physiotherapist or a medical specialist. PNs used motivational interviewing in patients who wanted to quit smoking or referred to a “Stop Smoking”- program. Patients were monitored 1–4 times a year by the PN and once a year by the GP to evaluate cardiovascular risk factors. With the multidisciplinary information system all involved disciplines had access to the patients’ data, facilitating communication between care givers and exchange of information.

#### Protocols

From the start of integrated CVRM care in 2010, a work protocol for the PN was available. The work protocol explains how to include patients in the CVRM care program and how to calculate the 10 year cardiovascular mortality risk based on the SCORE table [6]. In addition, the work protocol provides information about (i) a Minimal Intervention Strategy (MIS) for help with smoking cessation, (ii) how correct registration in Care2U should be done, (iii) when blood tests should be performed, (iiii) how to draw up an individual care plan and (iiiii) how to motivate patients to apply more self-management. Finally, the division of tasks between GP and PN is described. A protocol for correct blood pressure measurement was added in 2013.

#### Education

Annual education for GPs and PNs was organized by the care group and based on the most recent guidelines [6, 17]. PNs received additional training in motivational interviewing and data processing. In separate meetings for PNs only, they discussed complex case studies and shared problems on practice organization. In feedback meetings GPs and PNs discussed Care2U benchmark data on registration and outcomes.

#### Practice visitations

The care group started with practice visitations in 2015, carried out by care groups’ staff members to support practices with drawing up an annual practice plan and by formulating one or more areas in which a practice wanted to improve. From 2016 onwards, practice visitations were also used to discuss performance based on data from the quarterly reports.

#### Quarterly reports

The care group started with quarterly reports in 2016 that enabled practices to compare individual practice performance with care group performance. The care group established indicators for the prevalence, registration and outcomes of cardiovascular risk factors for participating practices. Standards were set for mean value, minimal norm and best practice, an often used method to compare individual performance with peer group performance [18]. Practices asking for support, having problems with organizing adequate CVRM care or performing below a minimal norm based on the care groups’ standards were prioritized for visitation. In 2015 the care group started with visiting 52 practices and in 2016, 2017 and 2018 respectively, 98, 102 and 117 practices were visited at least once.

#### LDL-cholesterol toolkit

The LDL-cholesterol toolkit introduced in 2017 comprised two parts: a part for care givers to inform patients on the use and necessity of lipid lowering medication (e.g. statins) and “tips and tricks” in case of impaired patient adherence or side effects. The other part was written information for patients.

### Ethical considerations

All methods used in this paper were carried out in accordance with the 2006 CVRM guidelines of the Dutch Society of General Practice and comply with the declaration of Helsinki. The study was approved by the ethics committee of the Julius Centre of Health Science and Primary Care, University Medical Centre Utrecht. Informed consent was given by patients participating in the CVRM care program. Due to legislation in the Netherlands, general practitioners are not allowed to register the ethnicity of their patients in the EHR.

### Data analysis

Data are presented as percentages and means with corresponding standard deviations, overall and in strata of primary and secondary prevention. Criteria for primary and secondary prevention are described in Table 1. All analyses were performed with IBM SPSS statistical software (version 22).

## Results

### Intervention studied

April 1^st^ 2010 27 practices with 8,456 eligible patients, started with the implementation of integrated CVRM care. January 1^st^ 2013, 137 practices with 38,675 eligible patients (mean age 67,8 years, 52,6% female, 43,6% ≥ 70 y, 33,4% with previous CVD) completed the implementation process with 149 practices and 48,397 participating patients (mean age 69,8 years, 53,1% female, 52,8% ≥ 70 years, 44,3% with previous CVD) participating in 2018. Because registration in Care2U was incomplete in 2010, annual results between 2011 and 2018 are shown. Baseline characteristics of participating patients eligible for primary and secondary prevention are given in Table 2.

**Table 2:** Characteristics of the study population (*)

Year	2011	2012	2013	2014	2015	2016	2017	2018
Registered patients in PoZoB care group (n)	378,099	402,623	406,119	416,433	422,296	401,077	407,661	416,648
Eligible for integrated CVRM care (n)	40,456	43,956	47,340	49,702	53,155	56,654	57,478	59,349
Participants (n)	34,628	38,675	39,503	42,551	45,138	48,222	46,400	48,397
Eligible but not participating	5858	5281	7,837	7,151	8,017	8,432	11,076	10,952
Participants eligible for primary prevention (%)	24,712(71,4)	25,772 (66,6)	25,559 (64,7)	26,802 (63,0)	27,913 (61,8)	28,639 (59,4)	26,029 (56,1)	26,935 (55,7)
Age (y) (SD)	66,3 (10,0)	66,6 (9,9)	66,7 (9,7)	66,9 (9,7)	67,2 (9,8)	67,5 (10,1)	67,3 (10,1)	67,6 (10,1)
Male (%)	43,9	43,6	43,5	43,7	43,6	44,0	43,6	44,0
≥ 70 years (%)	36,9	37,8	37,8	38,9	40,2	42,4	42,6	43,8
Mean LDL in mmol/l (SD)	3,34 (0,93)	3,31 (1,1)	3,25 (0,92)	3,13 (0,93)	2,96 (0,92)	2,97 (0,92)	2,88 (0,89)	2,62 (0,81)
Mean SBP in mm Hg (SD)	140,8 (19,8)	139,5 (16,5)	138,3 (15,7)	137,7 (15,9)	137,2 (15,3)	136,8 (15,0)	136,7 (15,1)	136,4 (14,9)
Smoking (%)	4,0	8,5	12,3	11,6	11,4	11,3	10,8	10,4
Non-smoking/stopped smoking (%)	55,2	65,5	78,5	78,9	80,4	80,5	81,1	81,7
LL medication prescribed (%)	22,8	32,4	35,6	37,5	41,1	42,9	49,9	49,6
BPL medication prescribed (%)	47,1	61,1	61,0	59,0	60,8	61,1	67,4	69,7
LDL ≤ 2,5 mmol/l (%)	15,0	19,6	22,7	27,9	35,9	34,0	39,5	51,8
SBP ≤ 140 mm Hg (%)	35,1	49,1	59,9	60,0	61,8	62,8	63,4	64,0
Non-smoking/stopped smoking, LDL ≤ 2,5 mmol/l and SBP ≤ 140 mm Hg (%)	3,7	7,5	11,4	14,2	18,5	18,8	20,8	29,5
Participants eligible for secondary prevention (%)	9,916 (28,6)	12,903 (33,4)	13,944 (35,3)	15,749 (37,0)	17,225 (38,2)	19,583 (40,6)	20,371 (43,9)	21,462 (44,3)
Age (y) (SD)	69,7 (11,8)	70,3 (11,3)	70,8 (10,8)	71,3 (10,9)	71,6 (10,8)	72,5 (10,9)	72,3 (10,8)	72,6 (10,7)
Male (%)	50,1	55,0	52,9	53,0	53,2	53,4	54,0	54,7
≥ 70 years (%)	53,3	55,3	57,2	58,7	59,7	62,4	62,8	64,1
Mean LDL in mmol/l (SD)	2,81 (0,91)	2,81 (0,90)	2,79 (0,90)	2,71 (0,90)	2,56 (0,89)	2,58 (0,88)	2,58 (0,87)	2,33 (0,82)
Mean SBP in mm Hg (SD)	138,1 (18,0)	137,1 (17,8)	136,5 (17,4)	135,9 (16,7)	136,1 (17,0)	135,6 (16,7)	135,2 (16,6)	135,0 (16,5)
Smoking (%)	4,2	9,8	14,4	13,5	13,4	12,8	12,2	12,2
Non-smoking/stopped smoking (%)	43,7	57,0	74,4	74,7	76,6	77,1	77,1	78,5
LL medication prescribed (%)	38,6	49,6	53,8	51,7	58,4	60,4	60,5	61,3
BPL medication prescribed (%)	49,6	58,8	62,8	60,8	65,8	66,9	66,7	68,6
LDL ≤ 2,5 mmol/l (%)	27,7	35,6	42,2	46,6	54,4	49,6	54,3	65,8
SBP ≤ 140 mm Hg (%)	32,9	48,3	62,6	61,8	62,2	63,2	63,4	64,5
Non-smoking/stopped smoking, LDL ≤ 2,5 mmol/l and SBP ≤ 140 mm Hg (%)	6,5	13,7	21,7	24,0	28,1	26,3	29,0	36,5

*Abbreviations*: *SD* Standard Deviation, *LL* medication: Lipid Lowering medication, *BPL* medication: Blood Pressure Lowering medication, *SBP* Systolic Blood Pressure, *LDL* LDL-cholesterol

(*) Reported proportions include all participating patients

### Registration

Registration in Care2U of LDL-cholesterol, SBP and smoking status improved significantly in 2011 and 2012 and remained stable in the subsequent years with missing data for LDL-cholesterol, SBP and smoking status of less than 10%. Registration data are given in Table 3.

**Table 3:** Registration of LDL-cholesterol*, SBP ** and smoking status between 2011 and 2018

	2011	2012	2013	2014	2015	2016	2017	2018
LDL-cholesterol *(%)	72,7	86,3	96,4	97,1	97,6	92,4	97,8	98,0
SBP** (%)	58,9	79,0	93,8	91,7	92,8	92,4	91,6	91,9
Smoking status (%)	55,9	71,6	90,1	89,7	91,1	91,0	91,8	91,6

LDL-cholesterol*: Low Density Lipoprotein-cholesterol. SBP**: Systolic Blood Pressure

### Risk factor trends in patients eligible for primary prevention

Cholesterol lowering prescriptions increased from 22,8% (2011) to 49,6% (2018). Blood pressure lowering prescriptions increased from 47,1% to 69,7%. Mean LDL-cholesterol decreased from 3,34 mmol/l to 2,62 mmol/l. Mean SBP decreased from 140,8 mmHg to 136,4 mmHg. The proportion of patients on target for LDL-cholesterol increased from 15,0% to 51,8%. The proportion of patients on target for SBP increased from 35,1% to 64,0%. The proportion of smoking patients decreased from 12,3% to 10,4% between 2013 and 2018. The proportion of non-smoking patients with LDL-cholesterol and SBP on target increased from 3,7% to 29,5%. A lower proportion of cholesterol lowering prescriptions in the primary prevention group resulted in higher mean LDL-cholesterol levels and a lower proportion of patients on target throughout all the years. On the other hand, despite similar proportions on target for SBP and blood pressure lowering prescriptions in both the primary and the secondary prevention group, mean SBP levels were slightly higher in the primary prevention group. A possible explanation is that PNs accept slightly elevated SBP values (140–150 mm Hg) more easily in patients without previous CVD.

### Risk factor trends for patients eligible for secondary prevention

**Fig. 1:**
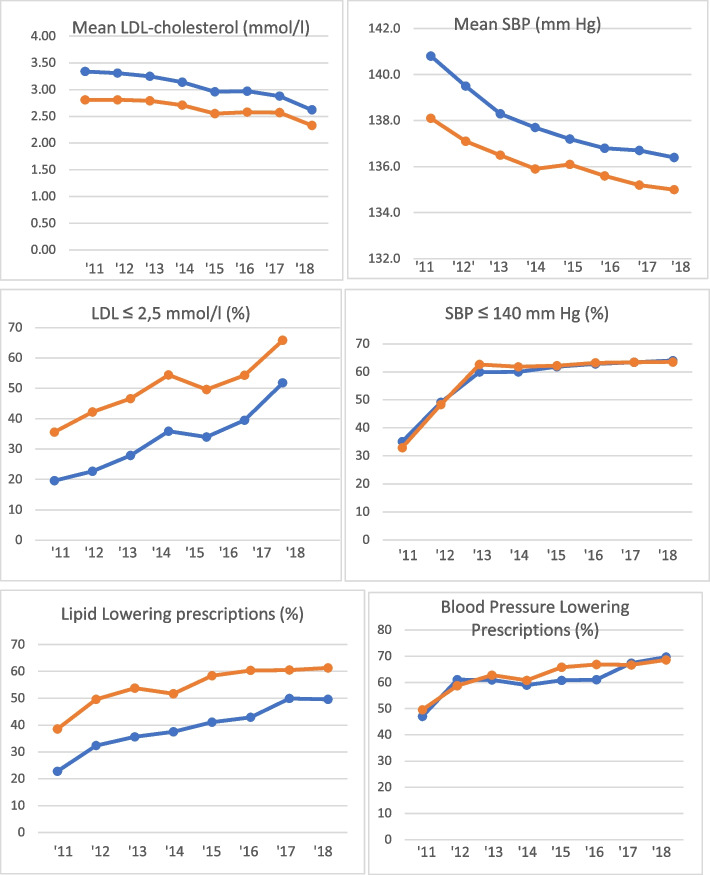
Mean LDL-cholesterol (mmol/l) and SBP (mm Hg), LDL-cholesterol and SBP on target (%) and Lipid Lowering and Blood Pressure Lowering prescriptions (%) between 2011 and 2018. Blue line: Primary prevention; Orange line: Secondary prevention. SBP: Systolic Blood Pressure; LDL: Low Density Lipoprotein

Cholesterol lowering prescriptions increased from 38,6% to 61,3%. Blood pressure lowering prescriptions increased from 49,6% to 68,6%. Mean LDL-cholesterol decreased from 2,81 mmol/l to 2,33 mmol/l. Mean SBP decreased from 138,1 mm Hg to 135,0 mm Hg. The proportion of patients on target for LDL-cholesterol increased from 27,7% to 65,8%. The proportion of patients on target for SBP increased from 32,9% to 64,5%. The proportion of smoking patients decreased from 14,4% to 12,2% between 2013 and 2018. The proportion of non-smoking patients with LDL-cholesterol and SBP on target increased from 6,5% to 36,5%. Figure 1 shows the course of mean LDL-cholesterol and mean SBP, the proportion of patients on target for LDL-cholesterol and SBP and the proportion of lipid lowering and blood pressure lowering prescriptions.

## Discussion

### Summary

The study showed that with offering comprehensive support, consisting of protocolized involvement of PNs, a multidisciplinary registration system, regular education and feedback, practice visitations, quarterly benchmark reports and a LDL-cholesterol toolkit, a considerable improvement in 3 important cardiovascular risk factors in patients on high risk for or with previous CVD was observed.

### Strengths and limitations

There is a number of strengths related to this study. First, the multidisciplinary information system (Care2U) allowed us to include a large study population with information from routine clinical practice. Second, all participating practices followed the same protocol for stepwise implementation and follow-up [16]. Third, data collection did not interfere with day-by-day practice, resulting in real life monitoring of a large number of patients at high risk of CVD in primary care. Fourth, data were collected over a period of 8 years which allowed us to visualize a clear development of both registration and outcomes.

There are also some limitations. First, because all affiliated practices started with programmatic CVRM care, this study contains no reference group. A causal relationship between care group support and outcomes therefore cannot be demonstrated. Long-term research, comparing integrated CVRM with care as usual would therefore be desirable. Second, some data on LDL-cholesterol, SBP and smoking status between 2010–2018 were missing, especially between 2010 and 2013. It remains uncertain whether this was solely due to technical problems, lack of time for the PN or not delivered care. However, the number of missing between 2013 and 2018 was generally well below 10%, and its impact on the results may be small [19]. Still, data registration could improve if PNs were given sufficient time to fulfill the administrative tasks. Third, although 24% of the patients has a non-Western European background, due to Dutch legislation it was not possible to compare the results within ethnic groups.

### Comparison with existing literature

To our knowledge this is among the first studies that evaluates comprehensive care group support and long-term outcomes of integrated CVRM care in the Netherlands. Comparison with other studies is difficult because the study design is often a randomized controlled trial (RCT) with a limited follow up period. Outcomes from RCTs comparing structured care delivered by PNs with care as usual show varying results. In two Dutch studies, PNs achieved equal or better results for the management of asthma, COPD, diabetes and cardiovascular risk factors after 1 year, compared to GP care [10, 20]. A nurse-led, multifaceted risk assessment and management program (RAMP) in hypertension patients reduced mean SBP and LDL-cholesterol and increased the proportion of patients on target for SBP and LDL-cholesterol as well as medication prescriptions after 1 year if compared to care delivered by GPs. Baseline SBP was 148.7 mm Hg, leaving a lot of room for improvement [21]. Another RCT, conducted in rural China, compared the delivery of a comprehensive intervention by family doctors (standardized medication, advice on both life style changes and medication adherence) with care as usual for hypertensive patients with or without diabetes. Increased prescribing rates of antihypertensive and lipid lowering medication were seen in the intervention group as well as significant reductions in smoking rates and salt intake but no differences in SBP and LDL-cholesterol after 1 year [22]. A recent meta-analysis by Stephen et al. showed significant improvements in nurse-led hypertension management in primary care after 6 months [23]. Van Bussel and co-workers, assessing a nurse-delivered multicomponent cardiovascular prevention program in general practice for patients between 70 and 78 years without cardiovascular disease found no differences in cardiovascular risk profile after a mean follow up period of 6 years, although the intervention reduced SBP and the proportion of smokers in the first 2 years of the study [24]. Marchal et al. assessed the effect of integrated CVRM care delivered by PNs compared to usual care offered by the GP in a randomized setting. They found no differences in SBP and LDL-cholesterol between the intervention and care as usual group after 1 year [25]. Yet, with mean baseline values for SBP and LDL-cholesterol of 136.7 mmHg and 2.8 mmol/l respectively, it seems difficult to improve within such a short period. It is known that implementation of a chronic care program and monitoring improvements is a lengthy process requiring regular adjustment [26]. With increasing awareness for CVRM and PNs participating in almost all general practices in the Netherlands, integrated CVRM care has become care as usual, thus reducing the contrast between study groups and partial explaining the lack of effectiveness in performed RCTs. This potential limitation could pave the way for long-term longitudinal cohort studies in the near future. Our study showed that in patients participating in integrated CVRM care between 2011 and 2018, we saw a substantial decrease in mean SBP, mean LDL-cholesterol and non-smoking participants, an increase in blood pressure lowering and lipid modifying prescriptions and an increase in patients on target for SBP and LDL-cholesterol. Finally, the proportion of non-smoking patients with SBP and LDL-cholesterol on target increased between 2011 and 2018 from 3,7% to 29,5% in patients eligible for primary prevention and from 6,5% to 36,5% in patients eligible for secondary prevention. Bager et al., described 8-year trends in blood pressure control, blood lipid control and smoking habits in hypertensive patients without CVD or diabetes, using a Swedish primary care register. They observed similar but smaller improvements in patients on target for systolic blood pressure and smoking, but very little improvements in patients on target for LDL cholesterol, leading to only 10% of all patients with 3 risk factors in control [27]. It is very possible that participation in an integrated CVRM care program led by a PN in our study contributed to better outcomes.Taking into account that with every mmol LDL reduction a reduction of 25% in major adverse cardiovascular events (MACE) can be achieved, a 10% reduction in MACE with every 5 mm Hg SBP reduction and a 32% reduction in MACE with smoking cessation [28–30], this implicates that for this large number of care group participants a considerable cardiovascular risk reduction is achieved. These findings indicate that with affiliation to a care group, adequate monitoring, subsequent treatment and long term follow up it is possible to realize clinical relevant improvements, resulting in a substantial risk reduction for CVD.

### Implications for research

In the Netherlands, care groups represent more than 15 million registered patients, which is 88% of the population. In 2020, with aggregated data from 86 primary care groups on integrated CVRM care it is possible to compare registration and outcomes to further improve results [31].

## Data Availability

Anonymized patient data derived from the multidisciplinary information system (Care2U) can be send by mail on request: g.smits@gmail.com.

## Abbreviations

PoZoB: Praktijkondersteuning Zuidoost Brabant
SGE: Stichting Gezondheidscentra Eindhoven
DOH: De Ondernemende Huisarts
CVRM: Cardiovascular risk management
GP: General Practitioner
CVD: Cardiovascular diseases
PN: Practice Nurse
EHR: Electronic Health Record
RCT: Randomized Controlled Trial
